# A dual RNA-seq analyses revealed dynamic arms race during the invasion of walnut by *Colletotrichum gloeosporioides*

**DOI:** 10.1186/s12870-024-05368-y

**Published:** 2024-07-10

**Authors:** Xichen Li, Yuhui Dong, Haiyi Yu, Jing Zhao, Fei Yang, Weichen Song, Changxi Wang, Jianning Liu, Qiang Liang, Yicheng Wang, Ke Qiang Yang, Hongcheng Fang

**Affiliations:** 1 College of Forestry, Shandong Agricultural University, Taian, Shandong Province China; 2 Mountain Tai Forest Ecosystem Research Station of State Forestry and Grassland Administration, Shandong Agricultural University, Taian, Shandong 271018 China; 3 State Forestry and Grassland Administration Key Laboratory of Silviculture in Downstream, Areas of the Yellow River, Taian, Shandong 271018 China; 4 State Key Laboratory of Crop Genetics and Germplasm Enhancement, Sanya Institute of Nanjing Agricultural University, Nanjing Agricultural University, Nanjing, 210095 China

**Keywords:** Walnut, *Colletotrichum gloeosporioides*, Dual transcriptomic, Invasion mechanisms, Response processes

## Abstract

**Background:**

Walnut anthracnose caused by *Colletotrichum gloeosporioides* seriously endangers the yield and quality of walnut, and has now become a catastrophic disease in the walnut industry. Therefore, understanding both pathogen invasion mechanisms and host response processes is crucial to defense against *C. gloeosporioides* infection.

**Results:**

Here, we investigated the mechanisms of interaction between walnut fruits (anthracnose-resistant F26 fruit bracts and anthracnose-susceptible F423 fruit bracts) and *C. gloeosporioides* at three infection time points (24hpi, 48hpi, and 72hpi) using a high-resolution time series dual transcriptomic analysis, characterizing the arms race between walnut and *C. gloeosporioides*. A total of 20,780 and 6670 differentially expressed genes (DEGs) were identified in walnut and *C. gloeosporioides* against 24hpi, respectively. Generous DEGs in walnut exhibited opposite expression patterns between F26 and F423, which indicated that different resistant materials exhibited different transcriptional responses to *C. gloeosporioides* during the infection process. KEGG functional enrichment analysis indicated that F26 displayed a broader response to *C. gloeosporioides* than F423. Meanwhile, the functional analysis of the *C. gloeosporioides* transcriptome was conducted and found that PHI, SignalP, CAZy, TCDB genes, the Fungal Zn (2)-Cys (6) binuclear cluster domain (PF00172.19) and the Cytochrome P450 (PF00067.23) were largely prominent in F26 fruit. These results suggested that *C. gloeosporioides* secreted some type of effector proteins in walnut fruit and appeared a different behavior based on the developmental stage of the walnut.

**Conclusions:**

Our present results shed light on the arms race process by which *C. gloeosporioides* attacked host and walnut against pathogen infection, laying the foundation for the green prevention of walnut anthracnose.

**Supplementary Information:**

The online version contains supplementary material available at 10.1186/s12870-024-05368-y.

## Background

*Colletotrichum gloeosporioides* is one of the most widely spread fungal pathogens that can infect over 470 host species and is the main pathogenic factor for post-harvest diseases in fruits including strawberry, mango, avocado, coffee, banana, and pomegranate [1, 2]. The *C. gloeosporioides* genome (approximately 55.6 Mb) was sequenced and assembled into 1241 scaffolds using the Illumina Hiseq 2000 sequencer [3]. Studies on *Colletotrichum* genomic and transcriptomic analyses have revealed that most *Colletotrichum* species adopt a hemibiotrophic lifestyle to complete the pathogenic process [4]. The germinating conidia form melanized appressoria to begin initial infection, which directly penetrate the epidermal cells. Following initial infection, primary hyphae, small molecular proteins, and absorbs secondary metabolites were produced to enter biotrophy stage. Finally, the infection switches to necrotrophy, that necrotrophic hyphae growing rapidly destroyed host tissues [2, 3]. The biotrophic stage is underpinned by secretion of effectors and the necrotrophic stage destroys plant cells through hydrolytic enzymes and toxins [4].

To cope with the invasion by pathogens, plants evolved a two-layered innate immune system: pathogen associated molecular pattern (PAMP) triggered immunity (PTI) and effector triggered immunity (ETI) [5, 6]. The response of ETI depends on the PTI, which PAMP recognized by receptors located on the surface of plant cells, transmits immune signals downstream through BIK1 (Botrytis induced kinase 1), MAPK cascade, CDPK, induces production of reactive oxygen species, hypersensitive response, expression of pathogen-related genes and finally triggers ETI. In turn, ETI enhances and restores plant resistance to pathogens by upregulating the PTI component [7, 8]. In recent years, the high-resolution temporal RNA-seq of both host plant and pathogen could be used to identify key genes associated with PTI and ETI and explore interaction patterns between pathogen and its host in depth, providing guidance for the green prevention strategies and resistant varieties.

The dual RNA-seq approach has been widely used to explore the interaction patterns between plants and pathogens [9, 10]. The maize- *Aspergillus flavus* regulatory network by dual RNA-seq identified new resistance genes in maize [11]. The potential genes highly correlated between the *Botrytis cinerea* and woodland strawberry (*Fragaria vesca*) were also identified through high-resolution time series dual transcriptomic analysis [12]. Similarly, a dual RNA-seq was performed to analyze the attack mechanisms of *Alternaria alternata* and the defense mechanisms of Chrysanthemum (*Chrysanthemum morifolium*) [13]. Meanwhile, interactions between hosts and *C. gloeosporioides* were also investigated by the dual RNA-seq such as strawberry [14] and red mango fruit [15].

Walnut (*Juglans regia* L.) is an important woody oil tree species, which kernels are rich in nutritional value and are beneficial to human health [16, 17]. But due to the lack of disease resistant varieties, walnut anthracnose caused by *C. gloeosporioides* has become a catastrophic disease in walnut production, which can lead to 30–50% yield loss [18, 19]. Previous studies have shown that the lifestyle transitions of *C. gloeosporioides* in infected walnut fruits is initial infection at 24 hpi, biotrophy at 48 hpi, and necrotrophy at 72 hpi [20]. Based on this lifestyle, a series of transcriptome and proteomic sequencing of walnut resistance to *C. gloeosporioides* were conducted, and multitudinous key genes and proteins related to walnut resistance to *C. gloeosporioides* were identified [21–23]. However, research on the interaction mechanism between walnut and *C. gloeosporioides* is still limited.

In this study, we conducted a dual RNA-Seq study to reveal the dynamics of the walnut-*C. gloeosporioides* interaction at infection time points (24hpi, 48hpi, and 72hpi). Differentially expressed genes (DEGs) analysis revealed that numerous DEGs exhibited opposite expression patterns between F26 and F423. KEGG functional enrichment analysis indicated that F26 displayed a broader response to *C. gloeosporioides* than F423. Synchronously, *C. gloeosporioides* secreted some type of effector proteins in walnut fruit and appeared a different behavior based on the developmental stage of the walnut. Our findings provided new insights into the green prevention of anthracnose and the breeding of walnut disease resistance varieties, thereby promoting the high-quality and healthy development of the walnut industry.

## Results

### Dual transcriptome sequencing of walnut and *C. gloeosporioides*

**Fig. 1:**
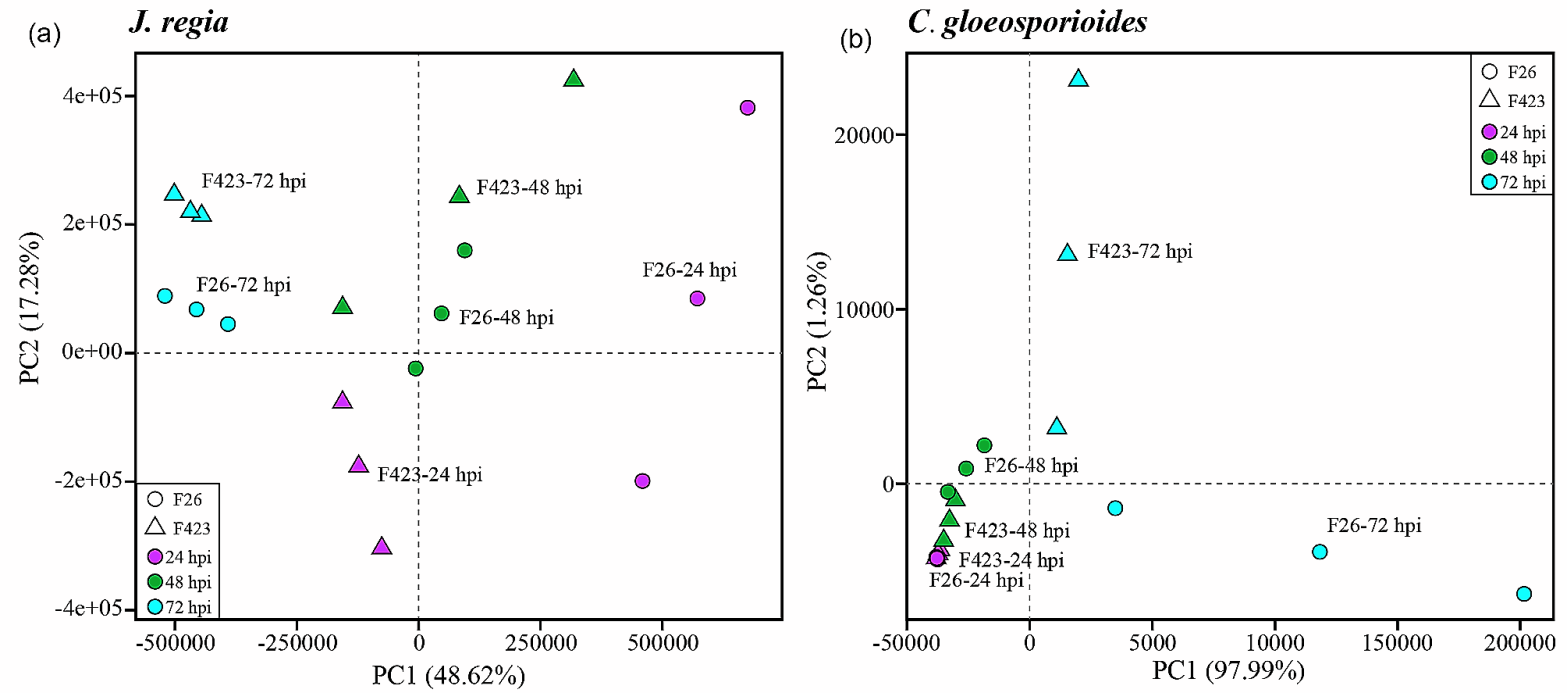
Principal component analysis (PCA) plots of the normalised count matrices of walnut and *C. gloeosporioides* represent the analysis of gene expression patterns. Generated by DESeq2 by differential expression analysis of disease resistant fruit F26 (○) and susceptible fruit F423 (∆). The labels indicate the time points (48 and 72hpi) after infestation for resistant as well as susceptible fruits

To reveal the dynamics of the walnut fruit-*C. gloeosporioides* interaction at different infection time points, we used the anthracnose-resistant (F26) and the anthracnose-susceptible (F423) walnut fruits as materials, and performed dual transcriptome sequencing on the infected tissue at 24, 48, and 72 hpi (hour post inoculated, hpi). The libraries were sequenced with an Illumina HiSeq 4000 platform. A total of 320.37 Gb clean data were obtained, with an average of 13.35 Gb per library (Table S1). The cumulative variance of PC1 and PC2 reached 65.9% in the principal component analyses (PCA), which unveiled that the samples based on the infection time point were clearly separated (Fig. 1a). For *C. gloeosporioides*, PC1 distinguished the samples between F26 and F423, while PC2 mainly divided the samples based on inoculation time points (Fig. 1b).

### DEGs analysis in the interaction between walnut and *C. gloeosporioides*

**Fig. 2:**
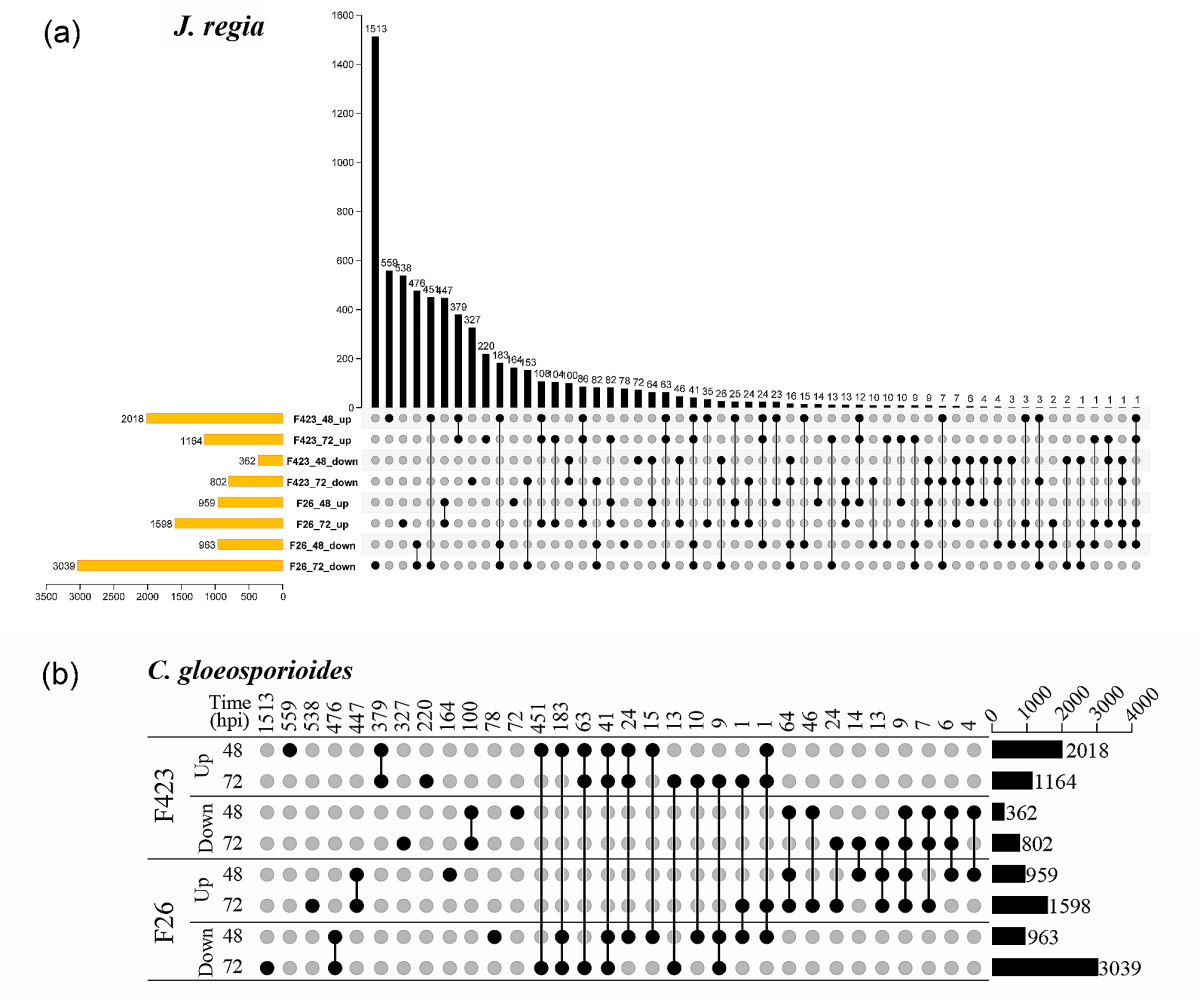
Data were obtained by comparing differentially expressed genes in walnut as well as *C. gloeosporioides* at different time points (48 and 72hpi). The highest number of DEGs in each group is indicated, and the dots and lines represent the common DEGs found between time points at each stage. (**a**). The differentially expressed genes at different time points for walnut disease-resistant fruit F26 as well as for disease-susceptible fruit F423. (**b**). Differentially expressed genes at different time points for *C. gloeosporioides*

To determine the responses of walnut fruit to *C. gloeosporioides* and to identify specific strategies used by the pathogen at specific times of infection, differentially expressed genes (DEGs) analysis was conducted. Walnut DEGs (P-adj ≤ 0.05) were detected by comparing the expression profiles of the host at each time point against 24 hpi for F26 and F423, respectively (Fig. 2a and Table S2). A total of 13,483 DEGs identified in F26 fruit and 14,958 in F423 fruit. The number of DEGs in F26 fruit continuously increased from 48 hpi to 72 hpi. In F423 fruit, the number of DEGs first increases and then decreases, reaching a peak at 48 hpi. Meanwhile, a large number of DEGs exhibited different expression patterns in F26 and F423.

In addition, we analyze how pathogens alter their transcriptional responses based on the initial time point of interaction. *C. gloeosporioides* DEGs were also detected by comparing the expression profiles of the fungus at each time point against 24 hpi for F26 and F423 fruit, respectively (Fig. 2b and Table S3). A total of 5025 DEGs were identified in F26 fruit and 3455 in F423 fruit. Similar to host DEGs, many upregulated DEGs in F26 exhibited opposite expression patterns in F423. These indicated that different resistant materials exhibited different transcriptional responses to *C. gloeosporioides* in during the infection process.

### Functional enrichment analysis of walnut DEGs for KEGG terms

**Fig. 3:**
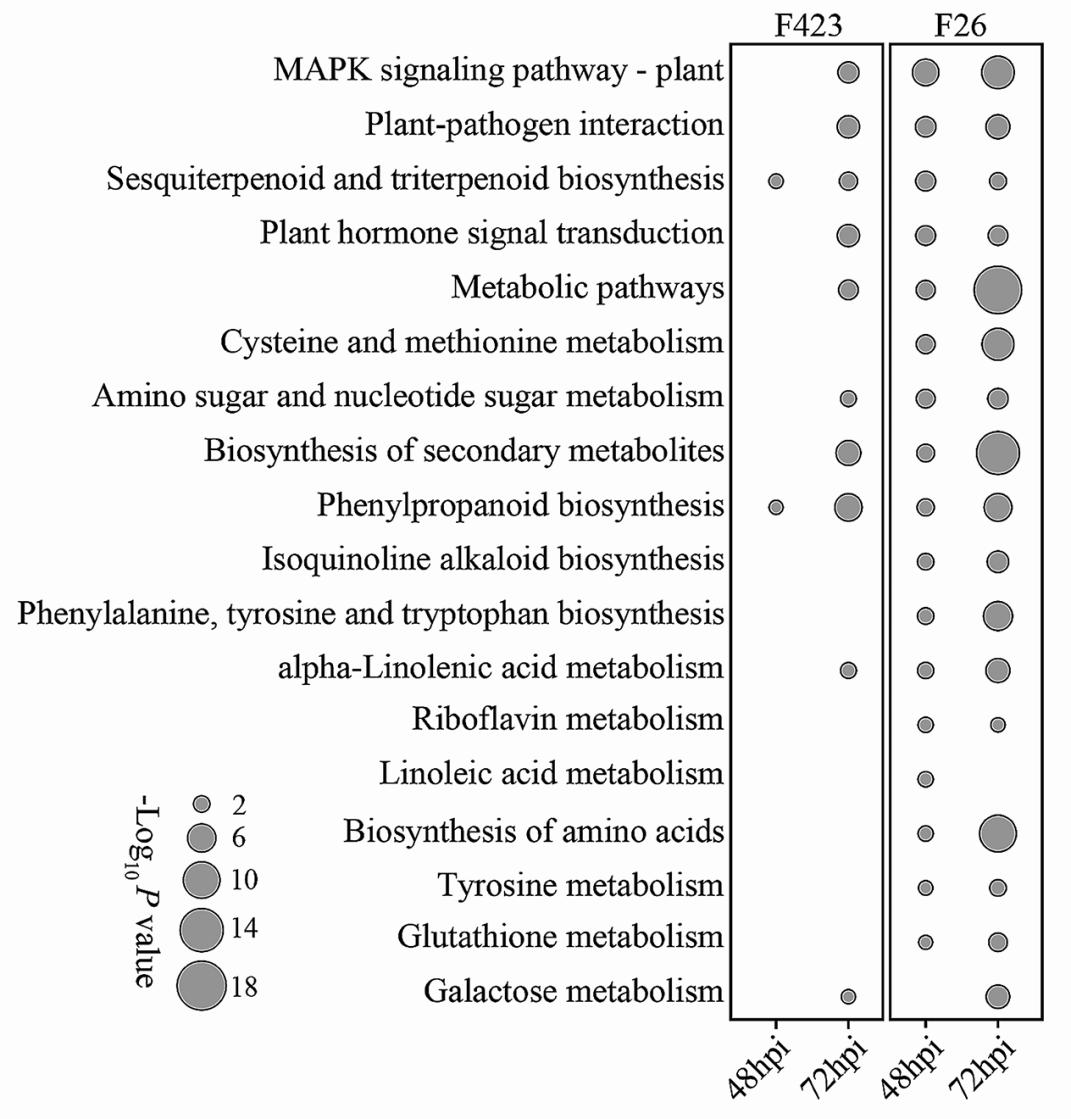
KEGG analysis of DEGs at two infection stages in F26 vs. F423. Metabolic pathways from the KEGG database were obtained in the four comparisons obtained in the differential expression analysis (disease resistance versus disease susceptibility). The dot size in the figure represents the logarithm of the inverted P-adj value obtained in the KEGG analysis versus the individual time points for both fruit

To study walnut metabolic pathways altered during *C. gloeosporioides* infection, we conducted a KEGG functional enrichment analysis of the walnut upregulated DEGs at 48hpi and 72hpi for F26 and F423 fruit (Table S4). KEGG terms that were significantly enriched in at least two out of the four comparisons between F26 and F423 fruit and the two time points were depicted (Fig. 3). Compared with the pathways in F423 fruit, multiple pathways were more evident in F26 fruit, which indicates that F26 fruit exhibited a strong stress response to the infection of *C. gloeosporioides*.

**Fig. 4:**
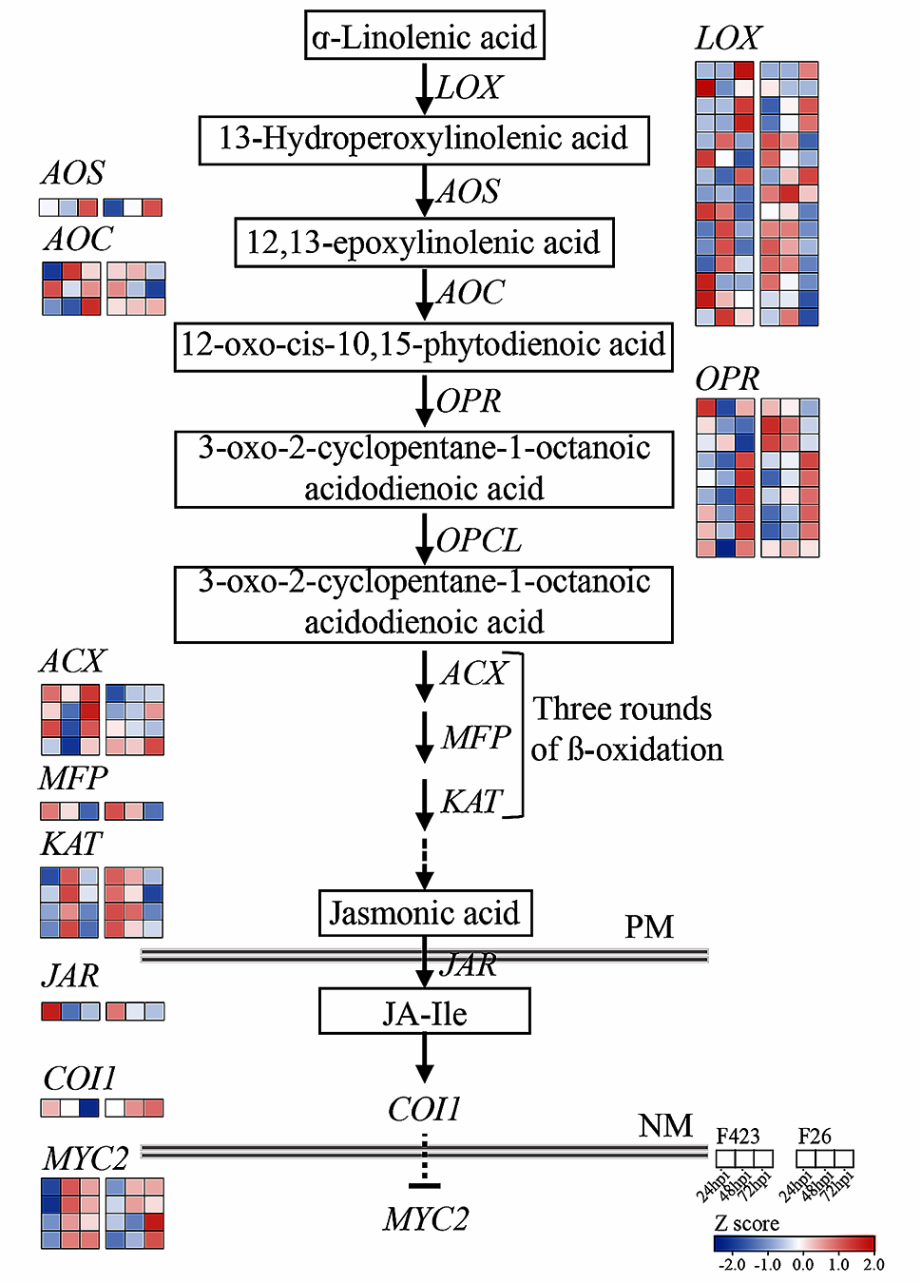
Activation of the jasmonic acid synthesis pathway in walnut fruit after inoculation with *C. gloeosporioides*. The scale colours of the heat map represent the intensity of significant expression changes, which are statistically derived from the intensity of expression at various time periods after inoculation of the samples

DEGs related to MAPK signaling pathway, plant-pathogen interaction and plant hormone signal transduction were enriched in F26 fruit at 48 hpi and 72 hpi, but it only appeared relevant in F423 fruit at 72 hpi. Pathways related to cysteine and methionine metabolism, isoquinoline alkaloid biosynthesis, and phenylalanine, tyrosine and tryptophan biosynthesis were only found to be enriched in F26 fruit at each time points. Given the enrichment of genes involved in plant hormone signaling transduction, a targeted analysis of jasmonic acid (JA) pathways was conducted. The transcriptional activation of JA biosynthesis is evident in walnut fruits, with a particular emphasis on the induction of multiple genes encoding the initial biosynthesis step, from lipoxygenase (LOX) to 12-oxygenated plant diene reductase (OPR). Related genes (*JAR*, *COI1*, and *MYC2*) in the later stage of biosynthetic pathways were also activated (Fig. 4).

### qRT‑PCR analyses of DEGs in the JA signaling pathway

**Fig. 5:**
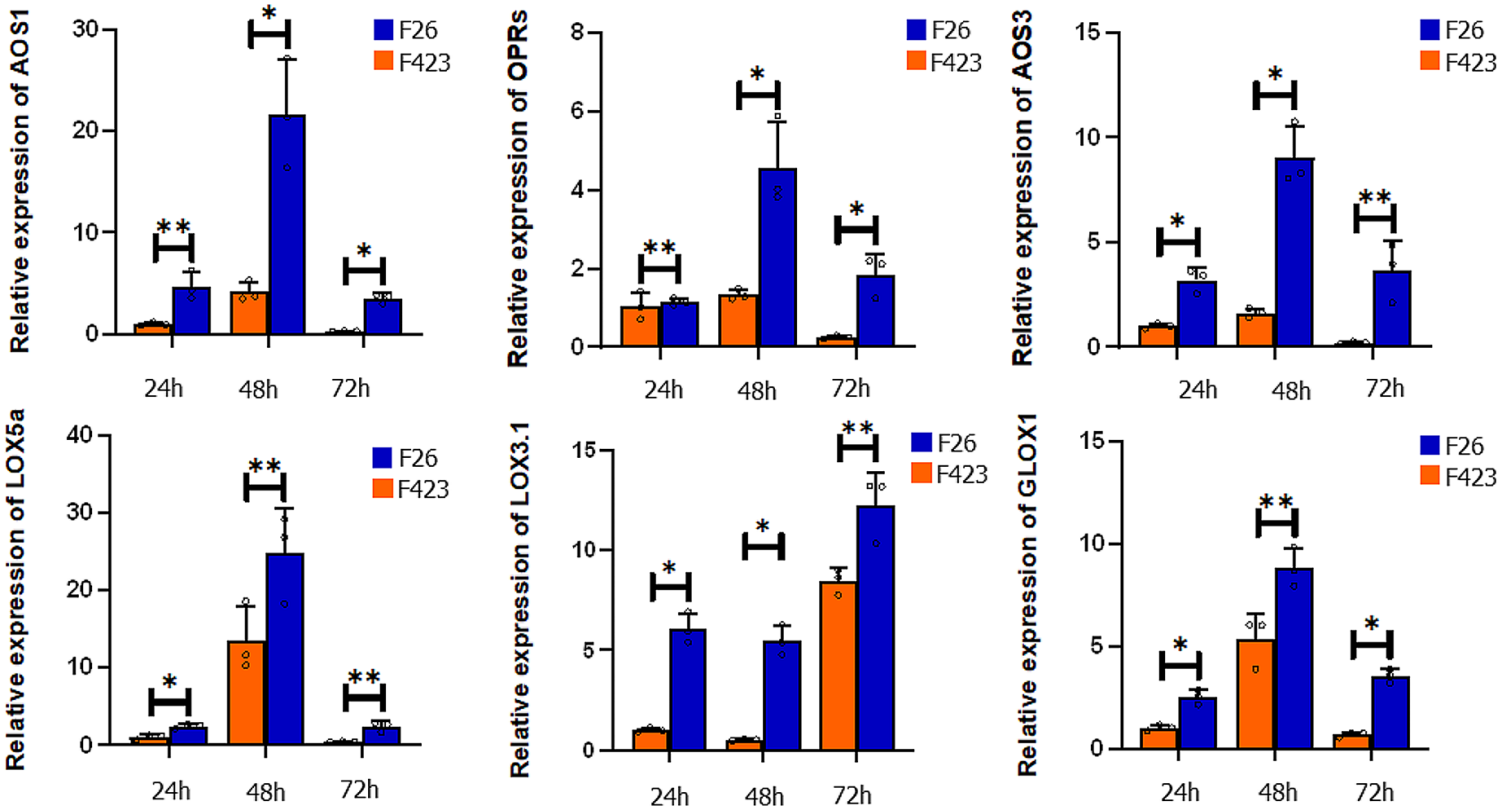
qRT‑PCR analyses of DEGs in the JA signaling pathway. Expression data were normalized against the data for the18S rRNA housekeeping gene and are presented as the mean ± standard error; *P < 0.05, **P < 0.01

To verify the expression profiles of DEGs in the JA signaling pathway, we selected six genes for qRT-PCR analysis (Table S5). The qRT-PCR results showed that the expression level of AOS1, OPRs, AOS3, LOX5a, LOX3.1, and GLOX1 in F26 was significantly higher than that in F423 (Fig. 5), which exhibited similar expression characteristics in the RNA-seq data (Table S2). The 18 S rRNA gene was used as a housekeeping gene, and the transcript abundance of six genes was normalized by comparison with the constitutive abundance of 18 S rRNA.

### The functional analysis of the *C. gloeosporioides* transcriptome

**Fig. 6:**
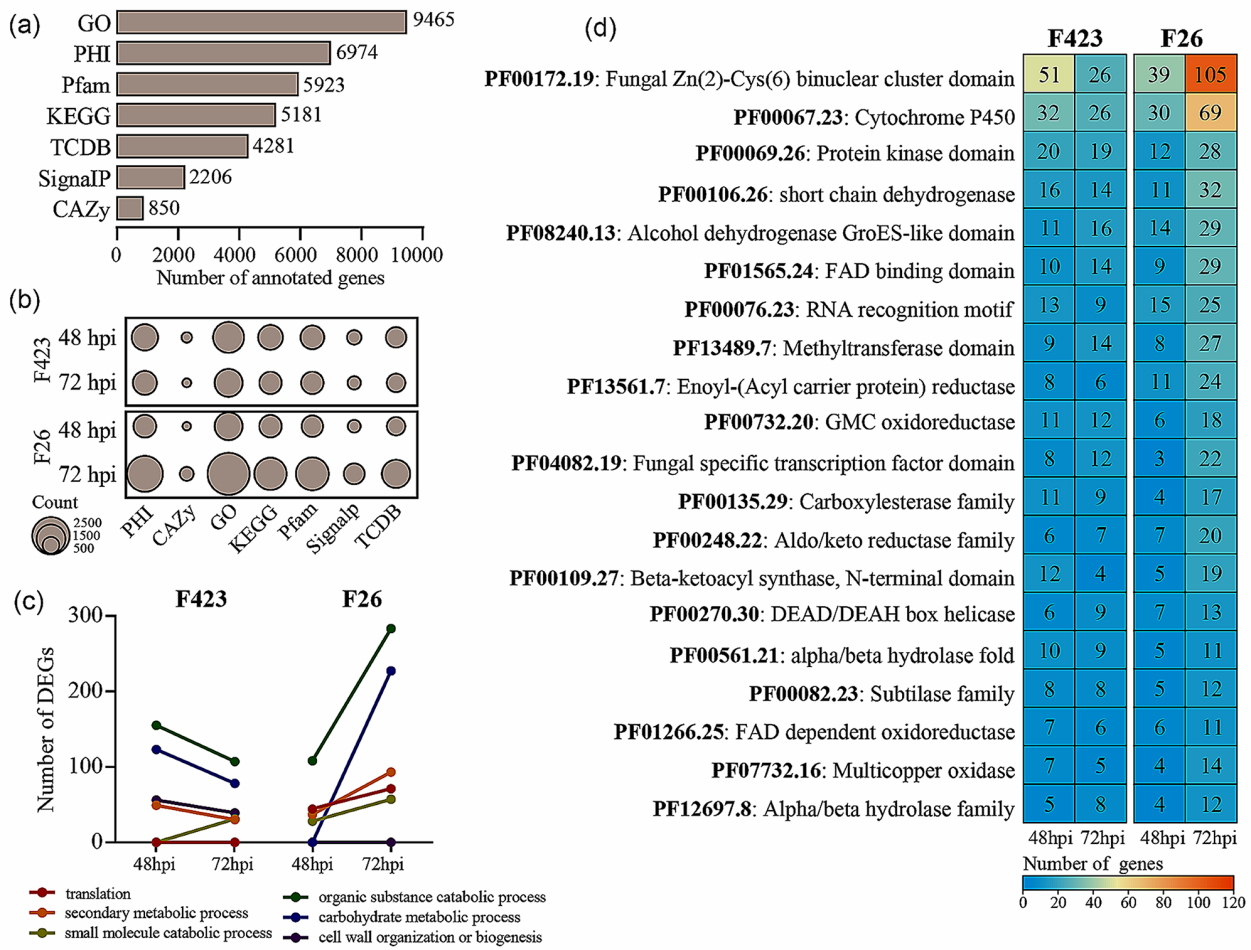
The annotated summary of the functions of *C. gloeosporioides*. (**a**). Functional annotations of transcripts obtained for all *C. gloeosporioides* types, each indicated by the number of DEGs. Includes GO gene ontology, PHI pathogen-host interactions, Pfam protein family database, TCDB transporter classification database, SignalP secretion signal peptide presence, and CAZy carbohydrate-active enzyme. (**b**). Enrichment of functional categories at 48 and 72 hpi in disease-resistant and disease-susceptible varieties of walnut. Dot size indicates the amount of differential gene expression. (**c**). Magnitude of response over time for walnut resistant and susceptible varieties to some correlation terms having the deg number of GO terms. Each colour represents a different GO term. (**d**). Enrichment of *C. gloeosporioides* in Pfam in the different phases obtained by analysing the DEGs of *C. gloeosporioides*. The colour scale of the heat map indicates the number of DEGs in each Pfam

To determine which fungal genes and functions involved in the interaction between walnut and *C. gloeosporioides*, the functional analysis of the pathogen transcriptome was conducted. Firstly, 15,368 de novo annotated transcripts were used for multiple functional categories, including pathogen-host interactions (PHI), membrane transport proteins (TCDB), proteins with signal peptides (SignalP), and carbohydrate-active enzymes (CAZy) (Fig. 6a and Table S7). Then, the enrichment analysis was performed on these major functional categories of upregulated DEGs during the infection process to obtain the overall situation of specific gene categories induced by pathogens in F26 and F423 fruits (Fig. 6b). In F26 and F423 fruit, these large categories were all enriched in *C. gloeosporioides* upregulated DEGs at both stages when compared to 24 hpi. Compared to F423 fruit, a significant abundance of PHI and TCDB genes in F26 fruit, especially at 72hpi.

Meanwhile, GO terms associated with fungal growth, virulence, and pathogenicity among *C. gloeosporioides* upregulated DEGs were identified at each host developmental stage (Fig. 6c). Except for the term on small molecule catabolic process, the number of *C. gloeosporioides* upregulated DEGs in F423 decreased from 48hpi to 72hpi, whereas in F26 fruit the upregulated DEGs increased along with infection time. In both fruits, *C. gloeosporioides* induced a high number of DEGs related to organic substance catabolic process (Fig. 6c). In addition, we also conducted the enrichments of Pfam domains using the *C. gloeosporioides* upregulated DEGs (Fig. 6d and Table S7). Consistent with previous results, Pfam categories were mainly enriched in F26 fruit at 72 hpi. Fungal Zn (2)-Cys(6) binuclear cluster domain (PF00172.19) and Cytochrome P450 (PF00067.23) were largely prominent in F26 fruit, especially at 72 hpi, where up to 105 and 69 genes were induced, respectively.

### Highly induced *C. gloeosporioides genes during inoculation*

To identify potential target genes for the control of *C. gloeosporioides*, the top five *C. gloeosporioides*-induced DEGs in F26 and F423 fruit were identified (Table 1). The top five DEGs were unique at different stages of different fruit types, which indicates that *C. gloeosporioides* displays a different behavior based on the developmental stage of the walnut. Strongly induced DEGs at 48 hpi of F423 fruit included 3-phenylpropionate/cinnamic acid dioxygenase (69,020,245), non-reducing polyketide synthase (69,019,196), and highly reducing polyketide synthase (69,017,928). A member of trihydrophobin (69,008,465) was highly expressed at 72 hpi in F423 fruit, alongside FAD-linked oxidoreductase (69,008,133), and Pyranose dehydrogenase 3 (69,017,633). In F26 fruit, alkanesulfonate monooxygenase (69,010,264), pectinesterase (69,018,227), and trihydrophobin (69,008,465) were highly expressed at 48 hpi. At 72 hpi, a putative mannosyltransferase KTR3 (69,011,099), highly reducing polyketide synthase FUM1 (69,013,575), initiation-specific alpha-1, 6-mannosyltransferase (69,012,672), and liver carboxylesterase 2 (69,007,734) were highly induced.

**Table 1:** The top five C. gloeosporioides-induced DEGs in F26 and F423 fruit

Accession	Log2FC	Description	Selected functional annotat
F423_48			
69020245	7.705429517	3-phenylpropionate/cinnamic acid dioxygenase subunit alpha	**PHI**: Ndo1(Penicillium_digitatum unaffected_pathogenicity) | **PFAM**: PF00355.27 (Rieske [2Fe-2S] domain)
69016720	7.067576044	-	**PFAM**: PF12937.8 (F-box-like)
69019196	6.985911457	Non-reducing polyketide synthase terA	**PHI**: GRS1(Fusarium_graminearum unaffected_pathogenicity) | **TCDB**: 4.C.1.1.18 (Hybrid PKS-NRPS synthetase)
69017928	6.436315361	Highly reducing polyketide synthase azaB, partial	**PHI**: ZtPks8(Zymoseptoria_tritici unaffected_pathogenicity) | **PFAM**: PF00109.27 (Beta-ketoacyl synthase, N-terminal domain) | **TCDB**: 4.C.1.1.18 (Hybrid PKS-NRPS synthetase)
69009558	6.436158799	-	-
**F423_72**			
69008465	10.1383071	Trihydrophobin	**PHI**: MHP1 (Magnaporthe_oryzae reduced_virulence) | **SignalP**: SP(Sec/SPI)
69008133	-9.273415962	FAD-linked oxidoreductase	**PHI**: CTB5 (Cercospora_nicotianae reduced_virulence) | **CAZy**: AA7 | **PFAM**: PF01565.24 (FAD binding domain) | **SignalP**: SP(Sec/SPI)
69017633	9.249228059	Pyranose dehydrogenase 3	**PHI**: Bab2_0277 (Brucella_abortus reduced_virulence) | **CAZy**: AA7 | PFAM: PF00732.20 (GMC oxidoreductase)
69012171	-9.063139906	-	-
69021390	-8.806836293	Carboxylic acid transporter protein-like	**PHI**: PHO84 (Cryptococcus_neoformans reduced_virulence) | **TCDB**: 2.A.1.12.3(Jen2p)
**F26_48**			
69010264	8.334060838	Alkanesulfonate monooxygenase	**PFAM**: PF00296.21 (Luciferase-like monooxygenase)
69018227	-7.627917749	Pectinesterase	**PHI**: Bcpme1 (Botrytis_cinerea unaffected_pathogenicity_reduced_virulence) | CAZy: CE8 | **SignalP**: SP(Sec/SPI)
69008465	7.53386327	Trihydrophobin	**PHI**: MHP1 (Magnaporthe_oryzae reduced_virulence) | **SignalP**: SP(Sec/SPI)
69017504	7.203913654	-	**SignalP**: SP(Sec/SPI)
69019020	7.070702123	-	**SignalP**: SP(Sec/SPI)
**F26_72**			
69011099	-10.66490679	putative mannosyltransferase KTR3	**PHI**: Ktr4 (Beauveria_bassiana reduced_virulence) | **CAZy**: GT15 | **SinalP**: SP (Sec/SPI)
69007215	-10.20829594	-	**CAZy**: PL9
69013575	-10.15384346	Highly reducing polyketide synthase FUM1	**PHI**: FUM1_(FUM5) (Fusarium_fujikuroi unaffected_pathogenicity) | **PFAM**: PF00109.27 (Beta-ketoacyl synthase, N-terminal domain) | **TCDB**: 4.C.1.1.18 (Hybrid PKS-NRPS synthetase)
69012672	-10.04646004	Initiation-specific alpha-1 6-mannosyltransferase	**PHI**: VdOCH1 (Verticillium_dahliae reduced_virulence) | **CAZy**: GT32
69007734	-10.04159497	Liver carboxylesterase 2	**PHI**: FgAP2s_(FGSG_02015) (Fusarium_graminearum reduced_virulence) | **PFAM**: PF00135.29 (Carboxylesterase family) | **SinalP**: SP (Sec/SPI) | **TCDB**: 8.A.117.1.3 (Neuroligin-4, X-linked)

## Discussion

Walnut anthracnose caused by *C. gloeosporioides* caused fruit necrosis, leaf scorching, and can cause 30-50% yield loss, which is a catastrophic disease in walnut production [24]. Currently, chemical control is still the main measure for controlling walnut anthracnose, which is restricted due to drug resistance and environmental pollution [25, 26]. In recent years, the rapid development of genomics research of walnut and transcriptome sequencing technology, have contributed to a more in-depth study of walnut resistance to *C.gloeosporioides*. A large number of potential resistance genes, proteins, and metabolic pathways have been identified [22, 23]. Meanwhile, the publication of the genome of *Colletotrichum* enabled conducting dual RNA-seq, in which transcriptomic changes in both host and pathogen are simultaneously analyzed [27]. A dual RNA-seq analysis of tomato and *C. gloeosporioides* revealed concurrent alteration in gene expression of both host and pathogen during infection [2]. Similar to this study, we performed the dual RNA-seq between walnut and *C. gloeosporioides* to understand the fungal attack strategies and walnut fruit defense response. In this study, the number of *C. gloeosporioides* DEGs in F26 fruit is significantly higher than that in F423 fruit, and most of the upregulated genes exhibited opposite expression patterns (Fig. 2). These indicated that the *C. gloeosporioides* exhibited different transcriptional responses in different resistant materials during the infection process. However, PCA results found no separation in the clustering of samples from F26-24hpi and F423-24hpi, indicating that the expression profile of *C. gloeosporioides* in the early stage is similar (Fig. 1).

*C. gloeosporioides* employed hemibiotrophic manners to complete the pathogenic process, including initial infection, biotrophy, and necrotrophy [2, 4]. The lifestyle transitions of *C. gloeosporioides* in the walnut fruit bracts have been observed, in which the penetration peg is induced at 24 hpi, the primary hyphae produced 48 hpi, and the fungus formed secondary and necrotrophic hyphae at 72 hpi [21]. Based on this, enrichment analysis of upregulated DEGs compared to 24hpi indicated a significant abundance of PHI, SignalP, CAZy and TCDB genes in walnut fruit (Fig. 6). Previous studies have shown that massive proteins with signal peptides and CAZy-encoding genes were involved in the infiltration, colonization, and spread of pathogens during infection [28].Meanwhile, the Fungal Zn(2)-Cys(6) binuclear cluster domain (PF00172.19) and Cytochrome P450 (PF00067.23) were largely prominent in F26 fruit. The fungal Zn(II)2Cys6 binuclear cluster family has proved to be an intrinsic feature of filamentous fungal growth and pathogenesis [29]. The CYP enzymes as membrane-bound hemoproteins play an important role in the detoxification of cellular metabolism and xenobiotics [30]. These results suggested that *C. gloeosporioides* may secrete some type of effector proteins in walnut fruit. In addition, the top five DEGs were identified and unique at different stages of different fruit types (Fig. 6), which indicates that *C. gloeosporioides* displays a different behavior based on the developmental stage of the walnut.

Once the host–pathogen interaction began, host triggered a rapid plant response at the initial infection stage [31, 32]. Recent studies have shown that F26 exhibits an earlier response to *C. gloeosporioides* infection at 24hpi compared to F423. Starting from 48hpi, the pathogen begins to infiltrate and shift towards the invasive necrotrophic stage, which is remained during later infection [20]. This study investigated the transcriptional response of walnut fruits at the late stage of infection by *C. gloeosporioides*, and the results showed that F26 fruit displayed a stronger transcriptional response to *C. gloeosporioides* infection. Meanwhile, KEGG functional enrichment analysis indicated that F26 fruit had a larger number of genes induced than F423 fruit at 48hpi, and pathways related to valine, leucine and isoleucine degradation associated with JA biosynthesis, plant hormone signal transduction pathway and MAPK signaling pathway were also significantly enriched in F26 (Fig. 4). JA are known to be involved in defense responses against necrotrophs by increasing the fruit antioxidant capacity [33, 34]. Both plant hormone signaling transduction pathway and MAPK signaling pathway were induced during PTI and ETI [35]. Overall, these findings suggested that inoculated F26 displayed a broader response to *C. gloeosporioides* than F423.

## Conclusions

Collectively, in this study, a dual RNA-Seq study between walnut and *C. gloeosporioides* was performed to explore the dynamic changes in transcriptional profiles of both host and pathogen simultaneously. We identified large amounts of DEGs in walnut-*C. gloeosporioides* interaction at different infection stages. Numerous DEGs in walnut exhibited opposite expression patterns between F26 and F423, which indicated that different resistant materials exhibited different transcriptional responses to *C. gloeosporioides* during the infection process. KEGG functional enrichment analysis indicated that F26 displayed a broader response to *C. gloeosporioides* than F423. Meanwhile, the functional analysis of the *C. gloeosporioides* transcriptome was conducted and found that PHI, SignalP, CAZy, TCDB genes, the Fungal Zn(2)-Cys (6) binuclear cluster domain (PF00172.19) and the Cytochrome P450 (PF00067.23) were largely prominent in F26 fruit. These results suggested that *C. gloeosporioides* secreted some type of effector proteins in walnut fruit and appeared a different behavior based on the developmental stage of the walnut. Our present results improve our understanding of the *C.gloeosporioides* infection mechanism and the defense mechanism of walnut, laying the foundation for the green prevention of walnut anthracnose.

## Methods

### Plant material and fungal culture

Walnut fruits were taken from the ‘B26’ asexual line (F26) and the ‘4–23’ asexual line (F423). The F26 walnut seedling scion was supplied by the Walnut Professional Farmers’ Cooperative of Dongliugang Village, Baishi Town, Wenshang County, Shandong Province, China. The F423 walnut tree was an intraspecific hybrid progeny of the parental varieties ‘Yuanlin’ and ‘Qinglin’ selected in 2002. According to our evaluation results of anthracnose resistance as previously described [18], the ‘B26’ fruit bracts were highly resistant to anthracnose, and the ‘4–23’ fruit bracts was highly susceptible to anthracnose.

The *C. gloeosporioides* strain ‘m9’ (GenBank ID: GU597322) was used in all experiments. The *C. gloeosporioides* isolates were placed on potato dextrose agar (PDA, potato 200 g/L, glucose 20 g/L, agar 17 g/L), medium and cultured until conidia were produced. The conidia of strain m9 were diluted with sterile water and prepared into spore suspension; the final concentration is 10^5^–10^6^ conidia/ml.

### Fruit inoculations

Isolated walnut fruits were perforated and inoculated according to the previous method [36]. The healthy fruits from the east, south, and west directions of each tested individual tree were selected at 90 days after flowering, and the samples were disinfected with 0.6% sodium hypochlorite and washed with sterile water. Each fruit was inoculated with 20 µL of spore suspension at a concentration of 10^6^ conidia mL^− 1^ on the fruit surface. Fruits were placed in a constant-temperature incubator at 25 °C and cultured in the dark. Samples of the inoculation site were collected at 24, 48, and 72 hpi, then snap-frozen with liquid nitrogen and stored at -80 °C for transcriptome sequencing. Take three independent samples as biological replicates at each infection time.

### RNA extraction, cDNA libraries preparation, and RNA sequencing

Frozen samples of plant fruits were ground in a mortar and pestle. Plant and fungal RNA was extracted using the Thermo Gene JET Plant RNA Purification Mini Kit (Thermo Fisher Scientific Inc., USA). This was followed by gel electrophoresis on agarose gels stained with nucleic acid dyes to determine degradation.

The quality of the RNA was examined using an Agilent Bioanalyzer RNA Nano chip Bioanalyzer (Agilent Technologies) to obtain an RNA Integrity number (RIN) over 7.Poly (A) selection and rRNA depletion are first performed, after which mRNA was purified and fragmented using fragmentation buffer (Thermo Fisher Scientific Inc, USA). RNA fragments were reverse transcribed using random hexamer primers. The second strand cDNA was subsequently synthesised using DNA polymerase I, dNTPs and RNase H. After completion of end repair, A-tailing and index ligation, the product was purified and amplified using the QiaQuick PCR extraction kit to form the final cDNA library. The RNA libraries of the test samples were sequenced on an Illumina HiSeq 4000 platform (Illumina, USA), generating 2 × 150 bp pairs of end-to-end sequencing reads.

### Data analysis and differential expression gene analysis

The raw data obtained from sequencing was quality trimmed and adapter clipped by Trimmomatic v. 0.39. The resulting high-quality data was mapped to the *Colletotrichum gloeosporioides* (v. NFU_CgLc1_1.0) [37] and *Juglans regia* genome assembly Walnut 2.0 (genome accession GCF_001411555.2) genome using HiSAT2 v. 2.2.1. Gene abundance was next quantified using feature Count v. 2.0.3. Differentially expressed genes (DEGs) in walnut fruit as well as *C. gloeosporioides* were obtained by comparing the expression profiles at 48hpi and 72hpi against 24 hpi using DESeq2 v. 1.34.0. After assessing the significance of the differences, genes, and proteins with a p-value of ≤ 0.05 and |log2foldchange| ≥ 1 were designated as differentially expressed genes and proteins. Principal coordinate analysis of gene expression was performed using the vegan v. 2.6-4 package in R v. 4.2.

### Functional annotation and analysis of walnut fruits and *C. gloeosporioides* genes

Transcripts were annotated with multiple databases. BLAST searches were carried out to the transporter classification database (TCDB, http://www.tcdb.org/) and the pathogen?host interactions database (PHI, http://www.phi-base.org/). Custom HMMER alignment results for HMM profiles from the protein families database (Pfam), the carbohydrate-active enzyme annotation database (dbCAN, http://csbl.bmb.uga.edu/dbCAN/), and the fungal peroxidases database (fPox, http://peroxidase.riceblast.snu.ac.kr/) were similarly included. The presence of secretion signal peptides was evaluated for all genes in the transcriptome using SignalP v.4.0 (ref. 50). Gene ontology (GO) terms were obtained by aligning protein sequences against UniProt database using BLASTP v. 2.10.1+. An e-value of 1e-5 was used as the cutoff value across all methods described (Please rewrite it to avoid duplication). TB tools carried out analysis of GO and KEGG categories enrichment with whole-genome gene sets as background and a q value ? 0.05 as statistically significant.

### Electronic supplementary material

Below is the link to the electronic supplementary material.

Supplementary Material 1

Supplementary Material 2

Supplementary Material 3

Supplementary Material 4

Supplementary Material 5

Supplementary Material 6

Supplementary Material 7

## Data Availability

The RNA sequencing data are available via NCBI with BioProject accession PRJNA612972.
